# ToxId: an efficient algorithm to solve occlusions when tracking multiple animals

**DOI:** 10.1038/s41598-017-15104-2

**Published:** 2017-11-07

**Authors:** Alvaro Rodriguez, Hanqing Zhang, Jonatan Klaminder, Tomas Brodin, Magnus Andersson

**Affiliations:** 1 Department of Physics, Umeå University, 901 87 Umeå, Sweden; 2 Department of Ecology and Environmental Science, Umeå University, 901 87 Umeå, Sweden

## Abstract

Video analysis of animal behaviour is widely used in fields such as ecology, ecotoxicology, and evolutionary research. However, when tracking multiple animals, occlusion and crossing are problematic, especially when the identity of each individual needs to be preserved. We present a new algorithm, *ToxId*, which preserves the identity of multiple animals by linking trajectory segments using their intensity histogram and Hu-moments. We verify the performance and accuracy of our algorithm using video sequences with different animals and experimental conditions. The results show that our algorithm achieves state-of-the-art accuracy using an efficient approach without the need of learning processes, complex feature maps or knowledge of the animal shape. *ToxId* is also computationally efficient, has low memory requirements, and operates without accessing future or past frames.

## Introduction

**Figure 1:**
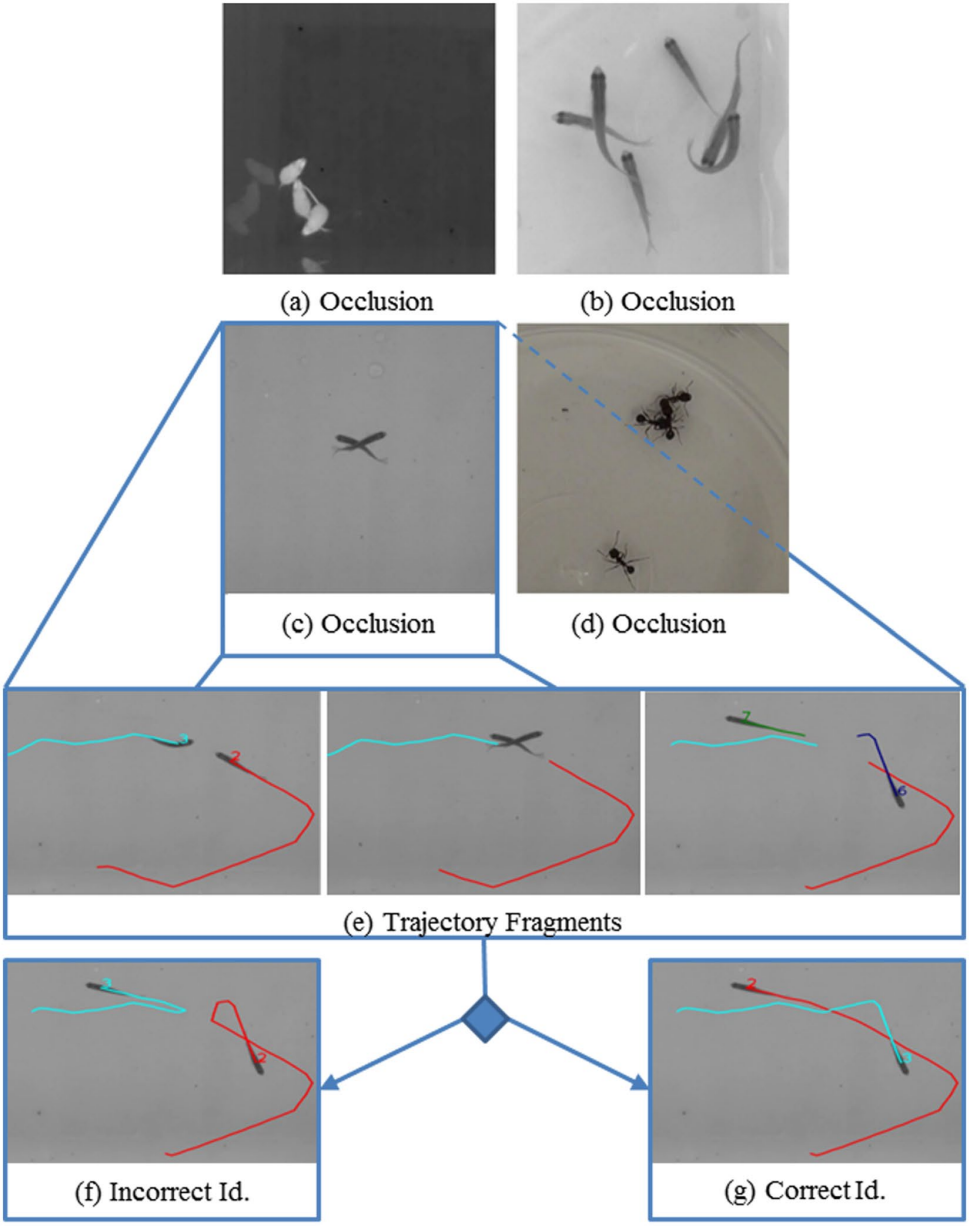
Occlusions. When animals cross or overlap, general tracking algorithms cannot preserve the identity of each individual; this scenario is called an occlusion. Occlusion examples are provided from the following datasets used in this study: (**a**) Mice3^26^, (**b**) Zebrafish11^18^, (**c**) Zebrafish5^6^, (**d**) Ant5^6^. For the occlusion shown in (**c**), trajectory fragments before and after the occlusion are shown in (**e**). To solve the occlusion, the correct fragments should be merged. An incorrect identification is shown in (**f**) and the correct identification is shown in (**g**).

Animal behaviour is important in many research fields such as ecology, medicine, neurology, ecotoxicology or evolutionary research^1^. In these fields, automatic tracking often relies on video tracking software to detect animal positions in controlled arenas^2,3^. While several methods provide a reliable tool for tracking one single individual^4,5^, preserving the identity of multiple individuals after an occlusion remains a challenging problem^1^, see Fig. 1 for an example. The complexity of this problem is illustrated in Pérez-Escudero *et al*.^6^, in a scenario where they solved correctly 99% of all crossings, but when considering error propagation only 11% of the animals were correctly identified after 2 minutes of tracking. Many state-of-the-art techniques, however, usually report a much lower accuracy. For example, in Itskovits *et al*.^4^ a multiple animal tracker system is proposed, which is able to solve only 77% of crossings between 2 animals.

To address the occlusion problem, some techniques tag the organisms with a visual marker to preserve their identity^7^. These solutions, however, may be invasive and are not applicable to the vast majority of animals. Other methods rely on improving the detection by using several cameras with different perspectives^8,9^. This technique adds complexity to the experimental setup and dramatically increases the amount of data generated. To improve detection and tracking, some techniques use a specific model of the animal body based on the head shape^10,11^, the body geometry^12–14^ or the symmetry axis^15^. Additionally, some authors discuss the use of features such as face properties^16^ or bilateral symmetry^17^. Nevertheless these methods can only be applied for animals geometrically compatible with the used model.

Other approaches to reduce occlusion problems rely on pattern recognition, matching specific texture maps^6^ or using convolutional neural networks^18^ to identify the animals. These techniques are computationally and memory expensive and require access to past and future frames (offline tracking). Thus, offline tracking is generally slow and cannot be applied in real-time, streaming applications, or other situations where only the current frame can be accessed. In these situations the use of an online tracking is required^19^.

To overcome the mentioned limitations - animal specific algorithms, slow algorithms that operate only offline, and low accuracy - we developed a new online algorithm called *ToxId*. *ToxId* can handle a large variety of animals and it does not use future or past frames, thus it can be used as a post processing stage in real-time applications. We show that *ToxId* achieves the same accuracy as the best state-of-the-art algorithm, also when considering error propagation. *ToxId* identifies animals in 97% of the frames and achieves this using the intensity histogram and the Hu-moments of detected animals. *ToxId* requires no training or complex configuration steps, it does not use features or characteristic fingerprint-like maps, and it requires significantly less memory than other algorithms^6,18^. *ToxId* is implemented in the free tracking software *ToxTrac* ^5^, which allows the user to extract locomotor activity such as; average speed, acceleration and distance traveled per time unit. In addition, it can also measure the time an organism spends near aquaria or terrarium walls. The software, the user manual and the documentation are available at https://toxtrac.sourceforge.io.

## Results and Discussion

*ToxId* can be included into the workflow of any online tracking algorithm and can detect and handle occlusions of multiple animals in an efficient way. When an occlusion takes place, *ToxId* cuts the tracked trajectories of the animals involved in the occlusion. The resulting trajectory fragments then needs to be fused in order to reconstruct the complete movement of each animal. To do this in a fast and reliable manner, *ToxId* extracts a set of visual features and positions of each animal in every frame and, at the end of the process, uses these features to fuse the fragments that have the highest probability to originate from the same individual. The features and positions of the animals are the only information needed to solve the occlusions, thus making the algorithm very computational efficient. We implemented *ToxId* in the free tracking software *ToxTrac* to run the analysis and we recommend not to use more than 10–20 animals in a single experiment to have reliable tracking.

To validate *ToxId*, we analyse multiple animals of different species under different experimental conditions and with different video resolutions and framerates. The main characteristics of these datasets, which are in total 48 minutes long and consist of approximately 80,000 frames, are summarized in Table 1. We first generate trajectory fragments from each video, where each fragment *f*_*J*_ is formed by a set of samples $\{{d}_{J0},\ldots ,{d}_{Jn}\}$, representing consecutive animal detections. Thereafter, we compare how *ToxId* merges trajectory fragments with a manually labelled ground truth. We define the validation metrics by counting the number of samples of correctly and incorrectly assigned fragments. The validation procedure is in short done as follows:

- We chose the first detected fragment of the animals as their reference identity.
- The fragments subsequently assigned by ToxId are marked as correct if they correspond to the same animal as the reference identity, and marked as incorrect otherwise. The remaining fragments are marked as not assigned.

**Table 1:** Dataset details. (*) dataset provided by the department of evolutionary biology and environmental studies from the University of Zurich. The original video can be downloaded at http://www.roborealm.com/tutorial/Blob_Tracking/index.php.

Dataset	Illumination	Video Resolution (pixel)	Frame Rate (fps)	Frames	Animal Size (pixel)	Animals
Ant5^6^	diffuse	1920 × 1080	25	15,000	925	5
Cockroach3	direct	2048 × 2048	25	10,000	15,500	3
Guppy2	direct	600 × 588	15	2,000	75	2
Mice3^26^	infrared	320 × 240	30	10,000	200	3
Waterlouse5	backlight	936 × 952	25	20,000	140	5
WingedAnt7*	direct	926 × 882	60	4,500	210	7
Zebrafish5^6^	diffuse	1506 × 1078	32	15,000	560	5
Zebrafish11^18^	direct	2048 × 2048	50	3,300	1,500	11

To quantify the algorithm performance we then use the following classification; the Identity Error Rate (IER) defined in equation (1), the Correct Fragment Rate (CFR) defined in equation (2), and the Correct Sample Rate (CSR) defined in equation (3).

1 $${\rm{IER}}=\frac{{\rm{IF}}}{\mathrm{Minutes}\times \mathrm{Animals}},$$

2 $${\rm{CFR}}=\frac{{\rm{CF}}}{\mathrm{CF}+\mathrm{IF}+\mathrm{NF}},$$

3 $${\rm{CSR}}=\frac{|{\rm{CF}}|}{|{\rm{CF}}|+|{\rm{IF}}|+|{\rm{NF}}|},$$

CF, IF, NF represent the correct, incorrect and unassigned fragments; and $|{\rm{CF}}|,|{\rm{IF}}|,|{\rm{NF}}|$ represent the corresponding number of samples. We discard short fragments without a sufficient number of samples (less than 25), and for the IER, we discard fragments that are shorter than one second.

**Figure 2:**
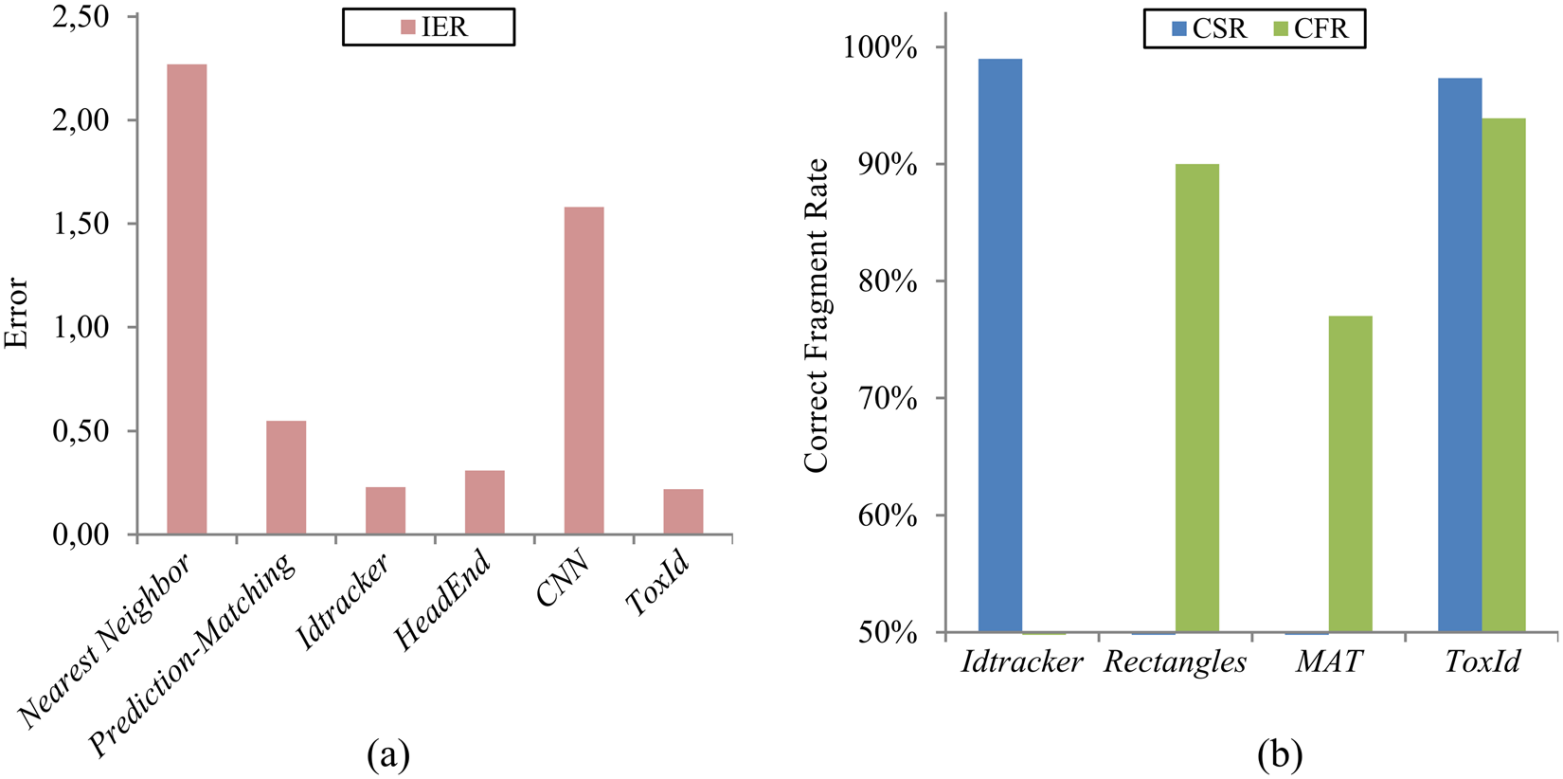
Algorithm comparison. (**a**) Identity Error Rate (IER) for the *Nearest-Neighbor* ^20^, *Prediction-Matching* ^11^, *Idtracker* ^6^, *HeadEnd* ^10^, *CNN* ^18^ and *ToxId* algorithms. (**b**) Correct Sample Rate (CSR) and Correct Fragment Rate (CFR) for the *Idtracker* ^6^, *Rectangles* ^14^, *MAT* ^4^ and *ToxId* algorithms.

A direct quantitative comparison with most published algorithms is not possible since different algorithms create the trajectory fragments differently. For example, tracking small features of the animal head will result in less occlusions than tracking the whole body, regardless of the occlusion solving strategy. Additionally, different algorithms cannot usually be applied to the same animals (restrictions in the animal shape) or to the same video (restrictions in resolution due to memory requirements). We therefore compare the performance of different techniques using the reported CSR when possible and the CFR or IER in other cases (Fig. 2).

Note that we use more strict CFR and ER definitions for *ToxId* than for other techniques. The reason for this is that other techniques do not consider error propagation. Thus, for *ToxId* a fragment is marked as correct if it is assigned to the original animal, while the compared techniques count correct and incorrect identifications independently for each crossing. In other words, we count identifications as incorrect if the same animal is identified before and after the crossing but the identity before the crossing was incorrect due to a previous error, other techniques will however count this as correct.

*ToxId* obtains a CSR of 97%, a CFR of 94% and an IER of 0.22 (Table 2). This is in line with the best published algorithm, *Idtracker* ^6^, which results are discussed in the next paragraph. *ToxId* achieves very good results when processing fish and aquatic invertebrates (e.g. waterlouse), but does not perform equally well with ants and cockroaches. Thus, if we consider these datasets separately (Ant5, Cockroach3 and WingedAnt7) *ToxId* obtains a CSR of 94%, a RCF of 85% and an IER of 0.40. For the other datasets *ToxId* obtains a CSR of 99%, a CFR of 96% and a IER of 0.12. We explain this difference by the complex orientations and deformations of ant and cockroach bodies combined with a high similarity between individuals. We also find that *ToxId* achieves a very good accuracy for a wide range of video resolutions and animal sizes, regardless of the illumination conditions, as long as conditions are uniform and constant. Video sequences with backlight illumination and opaque organisms of the same size are slightly more problematic to track since this illumination type hides texture differences of the organisms. To reproduce these results follow the instructions in the supplementary materials.

**Table 2:** Results of the analysis. (*) dataset provided by the department of evolutionary biology and environmental studies from the University of Zurich. The original video can be downloaded at http://www.roborealm.com/tutorial/Blob_Tracking/index.php.

Dataset	Total Samples	Trajectory Fragments	CSR	CFR	IER
Ant5^6^	68,850	149	94.5%	89.26%	0.28
Cockroach3	16,865	68	96.6%	87.30%	0.10
Guppy2	3,790	17	100.0%	100.00%	0.00
Mice3^26^	24,907	164	95.3%	92.55%	0.48
Waterlouse5	96,408	89	100.0%	100.00%	0.00
WingedAnts7*	30,095	38	91.1%	61.29%	0.81
Zebrafish5^6^	67,570	420	98.9%	95.66%	0.10
Zebrafish11^18^	31,662	227	99.6%	98.24%	0.00
**Total**	340,147	1,172	97.4%	94%	0.22

The *Idtracker* ^6^ occlusion handling algorithm is based on extracting feature maps that code the texture and act as characteristic fingerprints of each animal. This algorithm has been tested with several datasets of different animals, reporting a CSR of 99%. This approach, however, requires the access to past and future frames and cannot be applied for scenarios where online tracking is required^19^. Furthermore, *Idtracker* requires a huge number of samples for each individual to work (3,000 samples are recommended). Also, this method has been criticized for being limited to small animal groups and for requiring long video sequences to acquire enough reference frames^18^.

*Rectangles* ^14^ uses a chain of rectangles model to represent fish-like bodies. They obtained a mean CFR of 90% using two video datasets of 2,000 frames with 10 and 20 fish. These sequences are too short to measure the algorithm behaviour over time and do not consider error propagation.

*HeadEnd* ^11^ uses the head position and orientation of fish-like animals to predict their future positions. This method is tested using two short video datasets of 2,000 frames with 20 and 40 fishes. An average IER of 0.31 is achieved. They compared these results with: *Prediction-Matching* ^10^, with a IER of 0.55; N*earest-Neighbor* ^20^, with a IER of 2.27; and *Idtracker* ^6^, with a IER of 0.23. We conclude that the dataset used in this comparison was too small and no error propagation was considered. Therefore, these results have a limited validity and are difficult to compare to more thorough tests.

*CNN* ^18^ uses fish head detection algorithm and a Convolutional Neural Network to match the fish heads with the animals. Five video datasets (from 2,000 to 15,000 frames) with 5 to 25 fishes were used to test this algorithm and they reached an IER of 1.58. This algorithm is rather slow and cannot be applied for real-time processing, though it is reportedly able to process more individuals than *Idtracker* ^6^. However, it has the disadvantages of being one of the less accurate algorithms, only applicable to fish, and very inefficient, since it relies on the use of neural networks, which require a training stage and are generally slow. Additionally, they did not consider error propagation in their reported results.

Finally, the *MAT* ^4^ technique is an extension of the Kalman filter^21^ to predict the future position of multiple animals. They validated their algorithm by analysing two worms crossings, reporting a CFR of 77%. However, they did not consider error propagation in the reported results.

When considering proposed online algorithms that can handle occlusions^4,10,11,14^, we find that they usually can only be applied to a specific kind of animal^10,11,14^ or have significant accuracy problems when solving occlusions^4,20^. In contrast, offline techniques^6,18^ can in theory achieve a significantly higher accuracy than online techniques by accessing future frames of the sequence. *ToxId* however, achieves a CSR (97%) similar to the best technique, *Idtracker* ^6^ (99%), without accessing future or past frames. Furthermore, *Idtracker* also requires the use of complex texture maps with a heavy memory and computational cost. For example, to extract the texture maps of a sample of *n* pixels, *Idtracker* computational complexity^22^ is *O*(*n* ^2^)(has order of *n* ^2^ complexity), and the memory required is *O*(*n*). *ToxId* computational complexity is only *O*(*n*), and its required memory is *O*(1). This means that the *Idtracker* computational cost grows with a quadratic rate according to the pixel-number of the animal and its memory requirements grow linearly. *ToxId* computational cost grows only linearly with the pixel-number of the animal and its memory requirements do not grow.

## Conclusion

Quantitative analysis of animal behaviour is important in many fields^1^. These studies often generate a vast amount of data and many individuals need to be tracked simultaneously. Therefore, automatic techniques that can detect and track multiple organisms with accuracy, and at the same time are able preserve the identity of each individual, need to be developed. However, keeping the identity of the animals in a reliable way has proven problematic.

Current strategies to solve the identity problem use either an expensive strategy of texture analysis which requires access to past and future frames^6^, rely on features only valid for a particular kind of animal^11,14^, or combine both strategies^18^. Our new algorithm (*ToxId*) overcomes these limitations. *ToxId* requires no information of the shape of the animal, does not access to past or future frames and has low memory and computational costs.

We validated *ToxId* using 8 datasets with multiple animals of different species and in different experimental conditions by implementing *ToxId* in the latest version of the free tracking software, *ToxTrac*. The results show that, in most cases, *ToxId* achieves the same accuracy as the best state-of-the-art algorithm but with a significantly faster computational speed. We believe that *ToxId* represent a significant contribution in the study of multiple interacting organisms, as it overcomes some fundamental problems of current techniques.

## Methods

### Trajectory fragment creation

Animals are detected as dark moving objects in a constant bright background using a threshold intensity value defined by the user, who also introduces the number of animals in the experiment. The obtained objects or blobs are filtered by size to remove false-positives. During the detection, a number of image features representing each object are saved. Numerically, for a body *B* formed by a set of pixels {*p*}, we define the detection *d*_*B*_ as follows:

4 $${d}_{B}=\{C{M}_{B},{S}_{B},{H}_{B,kn},H{u}_{B},{t}_{B}\},$$

5 $${H}_{B,kn}=\{|\{x\in RO{I}_{B}|255\frac{k}{kn}\le x < 255\frac{k+1}{kn}\}|\}k=0,\mathrm{...},kn-1,$$

where *CM*_*B*_ is the center of mass of *B* ^23^; *S*_*B*_ is the number of pixels of *B* and represents its size; and ${H}_{B,kn}$ is the histogram of *B*, calculated by normalizing from 0 to 255 the rectangular area enclosing *B*, we denote this as ROI; *Hu*_*B*_ represent the Hu’s Seven Moments Invariants^24^ of *B*; and *t*_*B*_ represents the time of detection. Hu’s moment invariants are used to characterize patterns in images and they consists of six absolute orthogonal invariants and one skew orthogonal invariant that are calculated using weighted averages of the image intensity. The seven values characterize the image intensity distribution regardless of its location, scale and rotation. Therefore, it is very efficient for identifying rigid and moving objects regardless their orientation in the image.

To assign new detections *d*_*i*_ to existing trajectory fragments formed by sets of previous detections ${f}_{J}=\{{d}_{J0},\mathrm{...},{d}_{Jn}\}$, we use the Kalman^21^ algorithm. Thus, to every pair fragment–detection, we estimate the change in the size $({\rm{\Delta }}{S}_{i,Jn})$ and the Euclidean distance from the detection to the predicted fragment position the $({{\rm{cost}}}_{i,J})$ as follows:

6 $${\rm{\Delta }}{S}_{i,Jn}=\{\begin{array}{c}\frac{{S}_{i}-{S}_{Jn}}{{S}_{Jn}}:{S}_{Jn} < {S}_{i}\\ \frac{{S}_{Jn}-{S}_{i}}{{S}_{i}}:{S}_{i}\le {S}_{Jn}\end{array},$$

7 $${{\rm{cost}}}_{i,J}=\Vert {f}_{J}\,-\,{d}_{i}\Vert =\Vert C{M}_{Jn+1}\,-\,C{M}_{i}\Vert ,$$

Detections are assigned to fragments minimizing globally the costs with an Hungarian optimization algorithm^25^. To prevent assigning detection to a fragment incorrectly, the fragment is marked as inactive if the corresponding cost or change in size is higher than a certain limit, or if the fragment has not been assigned to any detection for a certain number of frames. We also mark fragments as inactive when two fragments are too close to the same detection.

### Fragment similarity

The goal of the algorithm is to connect the trajectory fragments that belong to the same individual; for simplicity, we note two fragments of the same individual with the equal sign. The first step is to construct a square matrix *IdMatrix* of size *N*x*N*, where *N* is the number of fragments and where the element $I{d}_{R,C}$ in the row *R* and column *C*, is a value representing the likelihood of ${f}_{R}={f}_{C}$. We initialize *IdMatrix* as an identity matrix and then set the values that are incompatible with each other (for example the fragments that coexist at a certain moment time) to 0. For the remaining values we will estimate a value $P({f}_{R},{f}_{C})$, this is expressed as follows:

8 $$I{d}_{R,C}=\{\begin{array}{cc}0, & {f}_{{\rm{R}}}\ne {f}_{{\rm{C}}}\\ 1 & {f}_{{\rm{R}}}={f}_{{\rm{C}}}\\ 0 < P({f}_{R},{f}_{C}) > 1, & \end{array},$$

where $P({f}_{R},{f}_{C})$ is the correlation of the similarity values *Sim* of *f*_*R*_ and *f*_*J*_ with all the fragments in the matrix. The idea behind this is that if two fragments belong to the same individual, not only their *Sim* value will be high, but also their *Sim* values will be similar regarding the remaining fragments. This can be expressed as follows:

9 $$P({f}_{R},{f}_{C})=R(\{Sim({f}_{R},{f}_{J})\},\{Sim({f}_{C},{f}_{J})\}):J=1,\mathrm{...},N,$$

where *R* is the Pearson correlation coefficient, and the similarity function between two fragments is defined as follows:

10 $$Sim({f}_{R},{f}_{J})=A\sum _{L=0}^{Ln-1}{w}_{L}{r}_{L}.$$

The first factor *A* represents a similarity amplitude equal to the correlation maximum of the histograms of the two fragments. It is expressed as,

11 $$A=\,\max \{R({H}_{Ri},{H}_{Ci})\}:{\rm{Ri}}=0,\mathrm{...},{\rm{Rn}},{\rm{Ci}}=0,\ldots ,{\rm{Cn}}.$$

The second factor of the similarity function, $\sum {w}_{L}{r}_{L}$, represents a normalized distribution of the histogram correlation. This distribution uses *L*_*n*_ levels and is weighted by a Gaussian function. This is expressed as,

12 $${w}_{L}={\rm{Gauss}}((\frac{L}{Ln}+\frac{L+1}{Ln})/2|\mu ,\sigma ),$$

13 $${r}_{L}=\frac{|\{x=R({H}_{Ri},{H}_{Ci})|\frac{L}{Ln}\le x < \frac{L+1}{Ln}\}|}{Rn\times Cn}:{\rm{Ri}}=0,\mathrm{...},{\rm{Rn}},{\rm{Ci}}=0,\mathrm{...},{\rm{Cn}},$$

where *μ* and *σ* are the mean and the standard deviation of the Gaussian curve, with default values of 1 and 0.05 respectively, and *Rn* × *Cn* is the total number of correlations. Thus, *r*_*L*_ is the rate of histogram correlations defined by *L*, and *w*_*L*_ is a weight assigned to this rate. In practice, we do not calculate the correlation of all samples, but only of those with a difference in shape and size below a certain value. We estimate the difference in shape of two samples as follows:

14 $$ShapeDiff({d}_{Ri},{d}_{Ci})=\sum _{h=1}^{7}|\frac{1}{H{{u}_{Ri}}^{h}}\,-\,\frac{1}{H{{u}_{Ci}}^{h}}|,$$

### Fragment assignment

**Figure 3:**
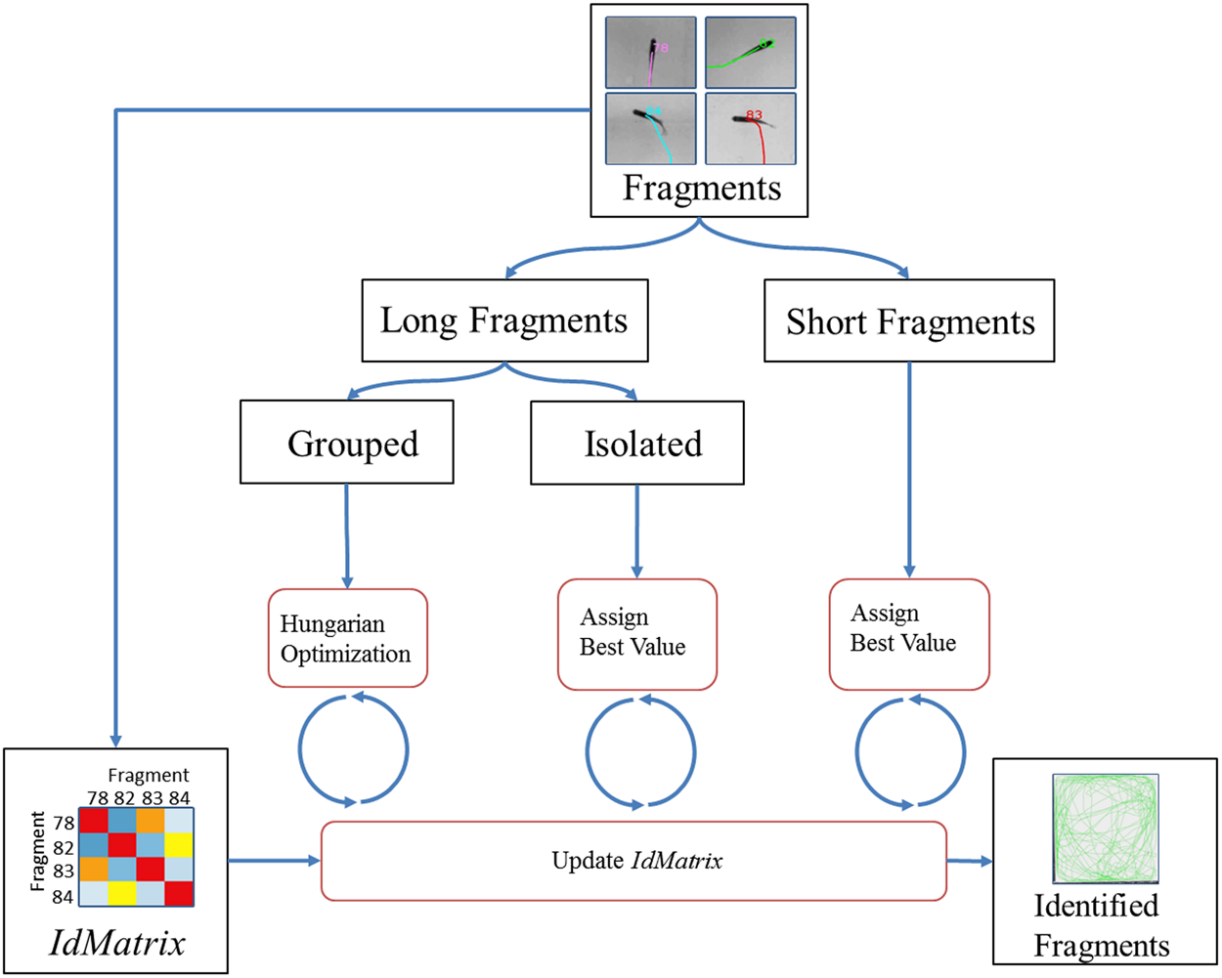
*ToxId* workflow. The matrix *idMatrix* contains the similarity between every two trajectory fragments. The fragments are divided between short and long according to the number of samples they contain. The assignment algorithm first uses a Hungarian optimization to assign groups of long fragments where all individuals are present. The remaining fragments are assigned one by one from higher to lower similarity.

To assign the different tracks to each other, the first step is to divide the fragments into long and short fragments according to a limit selected by the user (50 samples by default) and then choosing the groups of long fragments where all individuals are observed. Since this is an assignment problem with an optimal solution we use the classic Hungarian optimization algorithm^25^. To assign the remaining tracks, we iteratively select the best correlation value in the *idMatrix*, first with the long and then with the short tracks, till we reach a minimum correlation value selected by the user. With every assignment, we update the *idMatrix* and propagate the knowledge we obtain in each iteration, thus reducing the uncertainty for the remaining fragments. The fragment assignment algorithm workflow is shown in Fig. 3.

### Data availability

The *ToxId* algorithm has been implemented in the free tracking software *ToxTrac* ^5^, available at https://toxtrac.sourceforge.io v.2.70. The datasets analysed during the current study are available for public download at https://toxtrac.sourceforge.io and to reproduce the results in this work follow the steps provided in the Reproducing the results section in the supplementary materials. If you have problems accessing the files please contact the corresponding author.

### Statement

All experiments and methods were performed in accordance with relevant guidelines and regulations. All procedures and experimental protocols were conducted as stated and permitted by the Ethical Committee on Animal Experiments in Umeå (license no A41-12) and comply with current Swedish law.

## Electronic supplementary material

Supplementary materials
