# Supplementary material for: ToxId: an efficient algorithm to solve occlusions when tracking multiple animals
Source: Sci Rep. 2017 Nov 7;7:14774. doi: 10.1038/s41598-017-15104-2 (PMC5676683; doi:10.1038/s41598-017-15104-2)
Supplement: Supplementary file 1 — Supplementary materials [file 41598_2017_15104_MOESM1_ESM.pdf]

Monday, September 25, 2017

Supplementary materials

ToxId: an efficient algorithm to solve occlusions when tracking multiple animals

Alvaro Rodriquez[1], Hanqing Zhang[1], Jonatan Klaminder[2], Tomas Brodin[2], Magnus Andersson[1,*]

[1]Department of Physics, Umeå University, 901 87 Umeå, Sweden, [2]Department of Ecology and Environmental Science, Umeå University, 901 87 Umeå, Sweden

*Corresponding author: Magnus Andersson, Department of Physics, Umeå University, 901 87 Umeå, Sweden, Email: magnus.andersson@umu.se Phone: +46 90 786 6336

Keywords: animal behaviour; tracking; occlusion; computer vision;

1. Installing and running the software

We implemented the *ToxId* algorithm in the free tracking software *ToxTrac*[1], available at https://toxtrac.sourceforge.io.

ToxTrac requires Windows 7 or later, and is created for 64-bit hardware. We recommend a minimum of 8 GB of RAM memory and enough hard drive free space to handle all recorded video files. A 2.0+ GHz Quad core or higher is recommended for proper performance. A minimum screen resolution of 1280x800 is required to display properly the interface windows.

To install *ToxTrac*, it is only necessary to execute the .exe Windows installer file provided.

Visual Studio 2015 or Visual C++ x64 2015 Redistributable Packages (or newer) are required to run *ToxTrac*. The installer should automatically download and install this component if necessary. If the Visual C++ x64 2015 Redistributable Package installation fails, continue the installation, and if the program doesn't launch, install it manually from https://www.microsoft.com.

2. Quick user guide

ToxTrac supports a wide variety of .avi video files with any resolution and framerate. If the video is cut into several files add sequential numbers to the end of file names and placed them in the same folder.

Multiple video sequences can be analysed and multiple animals can be tracked in the same arena, however, we do not recommend using more than 10-20 animals in a single experiment.

Video resolution should be high enough so that the animal size is at least 50 pixels, and framerate should be high enough so the animal area in consecutive frames overlaps. Often, 25 fps is enough for most experiments.

ToxTrac will detect and track animals inside closed arenas. The tracking areas are uniform bright regions where the animals can be detected. If the arenas have dark corners or edges, exclude these from the tracking area in the program. Ideally, the background colour in the tracking areas should be homogeneous and significantly brighter than the animal. Aim for highest possible contrast.

The software can remove background objects, for example, objects much smaller than the animals can be easily filtered out by changing a parameter related to the size of the animals whereas static objects, of any size, can be removed by using the background subtraction algorithm. Recall that it is important to use a background as free of reflections or shadows as possible and to exclude the dark edges of the experimental setup from the background.

The software has a sophisticated calibration routine that only needs to be done once prior to a set of experiments. However, for this to work perfectly, the experimental setup should be isolated from external interference and light variations. It is important that the experimental setup keeps the same conditions and that it is not moved during an experiment or between experiments.

Finally, make sure you reduce strong shadows or reflections from the arena. This can be achieved by positioning the camera accurately, choosing the arena materials, and by using a diffuser or backlight illumination strategy. Additional details of how to build a setup and operate the program can be found in the complete User Guide located at: https://toxtrac.sourceforge.io.

3. Reproducing the results

The datasets analysed during the current study, with their corresponding ground truth and the *ToxTrac* project files are available for public download at https://toxtrac.sourceforge.io.

The results used in this study have been obtained using *ToxTrac* v.2.70. All results have been generated with the default configuration parameters for the *ToxId* algorithm. The detection and tracking parameters are different for each dataset, since the colour, the size, the video resolution other properties change from video to video. The ground truth may not be valid for different detection or tracking parameters, since the fragments of trajectories generated are dependent of the tracking. The configuration parameters used in the current study can be found in the project files available at https://toxtrac.sourceforge.io.

To reproduce the results in the current study perform the following steps:

- Install *ToxTrac* from https://toxtrac.sourceforge.io.
- To Download the data used in this study go to the folder "*Scientific Reports*" available at https://toxtrac.sourceforge.io or https://1drv.ms/f/s!AqgAEichDIhJpu83KecboJCnYnnIxg.
- Each data set is compressed into a zip-file. Download the preferable data and extract the zip file. Open the corresponding dataset project by clicking the *.tox* file, for example, *Zebrafish5.tox* from the windows explorer. *ToxTrac* should then launch and locate the video and the configuration files, and automatically load the necessary data. Under Results you will see the statistics of the analysis as well as the graphical outputs.
- To reanalyse the data just press the TRACK button. You can change the *ToxId* parameters to see the effects in the results. Do not change the detection or tracking parameters!
- To evaluate the obtained results copy the identification results located in the project folder, in the files *idMatrix_F.txt* and *idMatrix_Short_F.txt* in the corresponding table of the ground truth excel file *Zebrafish5.xlsx*.

References

1. Rodriguez, A. *et al.* ToxTrac: a fast and robust software for tracking organisms. *Methods Ecol. Evol.* **2017,** 1–5 (2017).
